# Five-year survival prognosis of young, middle-aged, and elderly adult female invasive breast cancer patients by clinical and lifestyle characteristics

**DOI:** 10.1007/s10549-024-07280-3

**Published:** 2024-03-25

**Authors:** Yu-Tung Teng, Yong Alison Wang, Yaa-Hui Dong, Jason J. Liu

**Affiliations:** 1 Institute of Public Health, National Yang Ming Chiao Tung University, No.155, Sec. 2, Linong St., Beitou District, Taipei, 112 Taiwan; 2 Koo Foundation Sun-Yat Sen Cancer Center, Taipei, Taiwan; 3 School of Medicine, National Yang Ming Chiao Tung University, Taipei, Taiwan; 4 Department of Pharmacy, National Yang Ming Chiao Tung University, Taipei, Taiwan

**Keywords:** Breast cancer, Five-year survival, Prognosis, Early-onset, Epidemiology

## Abstract

**Purpose:**

Early-onset breast cancer incidence has been increasing globally and in Taiwan. However, previous studies have not comprehensively examined how clinical and lifestyle characteristics influence the 5-year survival of breast cancer diagnosed at different stages of adulthood.

**Methods:**

We analyzed the Taiwan National Cancer Registry and Cause of Death datasets to understand how clinical factors (including tumor and treatment characteristics) and lifestyle factors (including body mass index, cigarette smoking, and alcohol consumption) were associated with the 5-year survival of 8471 young, 57,695 middle-aged, and 14,074 elderly female adult invasive breast cancer patients respectively diagnosed at age 20–39, 40–64, and ≥ 65 years between 2002 and 2015, with mortality follow-up to 2020. Poisson regression was used for obtaining the crude and adjusted 5-year survival risk ratios.

**Results:**

Clinical and lifestyle characteristics were distributed differently but had mostly similar direction of association with 5-year survival for the three age groups. Receiving any treatment was associated with better survival, especially for elderly patients. Being underweight at initial cancer treatment was associated with worse survival than having normal weight, especially for elderly patients. Current smokers had worse survival than never smokers for middle-aged and elderly patients. The 5-year breast cancer-specific survival was not significantly higher for those of age 45–49 years than 40–44 years, despite the recommended starting screening age is 45 years in Taiwan.

**Conclusion:**

Our findings contribute to the understanding of early-onset and later-onset female breast cancer characteristics and prognosis, which may inform surveillance and treatment strategies to achieve better breast cancer prognosis.

**Supplementary Information:**

The online version contains supplementary material available at 10.1007/s10549-024-07280-3.

## Introduction

Breast cancer is the most commonly diagnosed cancer worldwide in 2020, with an estimated 2.26 million new cases [1]. Breast cancer is also an important public health issue in Taiwan. According to the 2020 Taiwan Cancer Registry Annual Report, breast cancer was the second and third highest incident cancer among women 20–29 and ≥ 70 years of age respectively, and the highest incident cancer among women 30–69 years of age [2]. The burden of both premenopausal and postmenopausal breast cancer has been rising worldwide, and the incidence of early-onset breast cancer has been increasing in many countries [3]. In Taiwan, breast cancer incidence has been rising in the overall and young adult population. In addition, the 5-year survival of young adult breast cancer has been increasing in Taiwan [4]. It is therefore important to understand the characteristics and prognostic factors of both early-onset and later-onset breast cancer patients.

Previous studies have examined the characteristics of early-onset breast cancer [5, 6], but few studies have compared how clinical and lifestyle factors influence the prognosis of early-onset versus later-onset breast cancer. A Norwegian cancer registry study (*n* = 21,384) found women diagnosed at below 40 years of age had higher breast cancer-specific mortality compared with those diagnosed at age 50–69 years, particularly among those with luminal A-like tumors [7]. A study in the U.S. using cancer registry data from seven states (*n* = 5394) showed that there was no significant association between body mass index (BMI) and overall mortality among women with < 50 and 50–69 years of age at locoregional breast cancer diagnosis, but there was an inverse association among women diagnosed at 70 years of age or above [8]. Another study (*n* = 2265) has compared how smoking affected the prognosis of premenopausal versus postmenopausal breast cancer [9].

We used the nationwide Taiwan Cancer Registry data to conduct the largest study to our knowledge (*n* = 80,240) that compares the 5-year overall and breast cancer-specific survival of early-onset and later-onset breast cancer by tumor and treatment clinical characteristics. This is also the first study to understand how lifestyle factors including smoking status, alcohol consumption, and obesity are associated with breast cancer prognosis in an Asian population. We specifically compared how these clinical and lifestyle characteristics were associated with the 5-year overall and breast cancer-specific survival in young, middle-aged, and elderly female adult first primary invasive breast cancer patients respectively diagnosed at 20–39, 40–64, and ≥ 65 years of age. We also compared the breast cancer characteristics and causes of death across these three age at diagnosis groups. Findings from this study will help better inform early-onset and later-onset breast cancer patients of their prognosis, as well as contribute to efforts to improve the 5-year survival of breast cancer patients overall.

## Materials and methods

### Data source and study population

We conducted a nationwide retrospective cohort study using the Taiwan National Cancer Registry’s Annual Report and Long Form Datasets to obtain first primary invasive breast cancer diagnoses from 2002 to 2015, and the National Cause of Death Registry to determine the time and causes of deaths verified by death certificates from 2002 to 2020. Young, middle-aged, and elderly female adult breast cancer patients were respectively diagnosed at 20–39, 40–64, and ≥ 65 years of age.

**Fig. 1:**
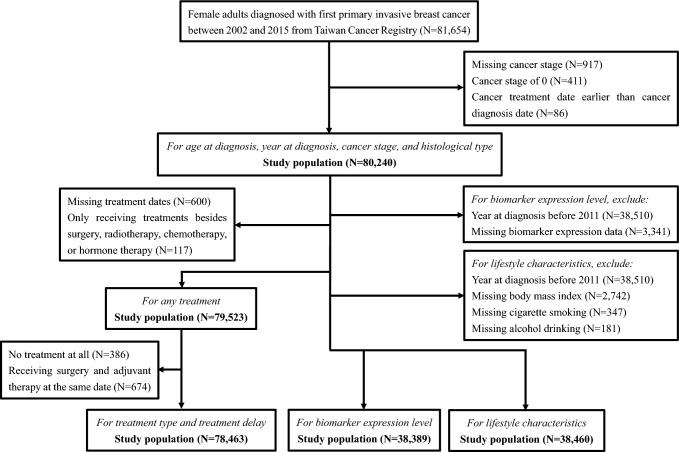
Flow chart for the selection of the study population

Figure 1 shows the selection of our analytic samples. We obtained a cohort of 80,240 breast cancer patients after excluding those with missing cancer stage (*n* = 917), stage 0 cancer (*n* = 411), or date of cancer treatment before diagnosis (*n* = 86). For the analyses of receiving any standard treatment, patients with missing treatment dates (*n* = 600) and those only receiving treatments besides surgery, radiotherapy, chemotherapy, or hormone therapy (*n* = 117) were excluded. For the analyses of treatment type and treatment delay, untreated patients (*n* = 386) and those receiving surgery and adjuvant therapy at the same date (*n* = 674) were excluded. Information on biomarker expression level, cigarette smoking, alcohol drinking, and BMI were only available at 2011 or later. For the analysis of biomarker expression level, patients who were diagnosed before 2011 (*n* = 38,510), or missing biomarker expression level (*n* = 3341) were excluded, resulting in an analytic sample of 38,389 patients. For the analysis involving BMI, cigarette smoking, and alcohol drinking, patients who were diagnosed before 2011 (*n* = 38,510), missing BMI (*n* = 2742), missing cigarette smoking (*n* = 347), or missing alcohol drinking (*n* = 181) information were excluded, resulting in an analytic sample of 38,460 patients.

This study was approved by the Institutional Review Board of National Yang Ming Chiao Tung University (Taipei, Taiwan).

### Variable definition

#### Independent variables

We examined clinical and lifestyle characteristics of the breast cancer patients as independent variables. Clinical characteristics included age at diagnosis, cancer stage, histological type, biomarker expression level, cancer treatment type, and treatment delay. Lifestyle characteristics included BMI, cigarette smoking, and alcohol drinking.

Cancer stage was determined according to the American Joint Committee on Cancer’s staging system. We used clinical stage information whenever it was available and pathological stage information if no clinical stage information was present. Histological type was determined using the morphology codes in the International Classification of Diseases for Oncology, 3rd Edition (ICD-O-3) [10], and we examined infiltrating ductal carcinoma (morphology code 8500), lobular carcinoma (morphology code 8520), mucinous adenocarcinoma (morphology code 8480), infiltrating ductal and lobular carcinoma (morphology code 8522), infiltrating duct mixed with other carcinomas (morphology code 8523), and all other histological types combined (all other ICD-O-3 breast cancer morphology codes). To define biomarker expression level, we used information on whether breast cancer cells had estrogen receptor (ER), progesterone receptor (PR), or human epidermal growth factor receptor 2 (HER2). HR+ meant tumor cells had ER or PR. HER2+ meant tumor cells had HER2 protein overexpression from immunohistochemistry and/or gene amplification from in situ hybridization. Participants receiving any cancer treatment were those who received surgery, chemotherapy, radiotherapy, or hormone therapy within five years after cancer diagnosis. Treatment delay was defined as the time interval from the date of breast cancer diagnosis to the date of the patient’s first cancer treatment, and was categorized as ≤ 30, 31–60, 61–90, or > 90 days [11].

For the lifestyle characteristics, BMI was measured before the first breast cancer treatment, and was categorized as underweight (< 18.5 kg/m^2^), normal (18.5-23.9 kg/m^2^), overweight (24-26.9 kg/m^2^), slightly obese (27-29.9 kg/m^2^), moderately obese (30-34.9 kg/m^2^), or severely obese (≥ 35 kg/m^2^), according to the cut-points of the Taiwan Ministry of Health and Welfare’s Health Promotion Administration [12]. Cigarette smoking status before breast cancer diagnosis was self-reported and categorized as never, former, or current. Alcohol drinking status before breast cancer diagnosis was self-reported and categorized as never, former, current casual drinking, or current habitual drinking. The cigarette smoking and alcohol drinking status categories were based on available classifications in the dataset.

#### Dependent variables

Five-year overall survival was defined as the percentage of patients who survived five years after first primary breast cancer diagnosis. For analyses involving treatment type, treatment delay, and BMI, 5-year overall survival was defined as the percentage of patients who survived 5 years after the first breast cancer treatment, as BMI was measured at cancer treatment. 5-year breast-cancer specific survival was defined as the percentage of breast cancer patients who did not die from breast cancer five years after cancer diagnosis or treatment.

#### Causes of death

To compare the causes of 5-year mortality among young, middle-aged, and elderly adult breast cancer patients, we classified the causes of death according to the International Statistical Classification of Diseases and Related Health Problems, 10th Edition [13].

### Statistical analysis

We used the chi-squared test to compare the distribution of clinical and lifestyle characteristics between young, middle-aged, and elderly adult breast cancer patients in Table 1. Poisson regression with robust variance was used to estimate the crude and confounding-adjusted 5-year survival risk ratios (RR) and their 95% confidence intervals (CI). For all adjusted analyses, we adjusted for year at diagnosis. For the adjusted analyses involving the three tumor characteristics variables (i.e., age at diagnosis, histological type, and cancer stage), we mutually adjusted for these variables. For the adjusted analyses involving biomarker expression level and any cancer treatment, we adjusted for the three tumor characteristics variables. For the adjusted analyses involving cancer treatment characteristics (i.e., treatment type and treatment delay), we mutually adjusted for these variables plus the three tumor characteristics variables. For the adjusted analyses involving lifestyle factors (i.e., BMI, cigarette smoking, and alcohol drinking), we mutually adjusted for these variables plus the three tumor characteristics variables. *P*-value < 0.05 was considered statistically significant. Analyses were conducted using SAS version 9.4.

**Table 1:** Clinical and lifestyle characteristics among young, middle-aged, and elderly adult female invasive breast cancer patients

Characteristics	All BC (N, %)	Young adult BC (N, %)	Middle-aged BC (N, %)	Elderly BC (N, %)
Age at diagnosis (years)				
20–39	8471 (10.6)			
40–64	57,695 (71.9)			
65+	14,074 (17.5)			
Year at diagnosis				
2002–2006	14,118 (17.6)	1853 (21.9)	10,187 (17.7)	2078 (14.7)
2007–2010	24,392 (30.4)	2617 (30.9)	17,740 (30.7)	4035 (28.7)
2011–2015	41,730 (52.0)	4001 (47.2)	29,768 (51.6)	7961 (56.6)
Cancer stage				
I	30,213 (37.6)	3053 (36.0)	22,750 (39.4)	4410 (31.3)
II	36,827 (45.9)	4202 (49.6)	25,849 (44.8)	6776 (48.2)
III	8150 (10.2)	842 (10.0)	5549 (9.6)	1759 (12.5)
IV	5050 (6.3)	374 (4.4)	3547 (6.2)	1129 (8.0)
Histological type				
Infiltrating ductal carcinoma	67,607 (84.3)	7146 (84.4)	49,043 (85.0)	11,418 (81.1)
Lobular carcinoma	3263 (4.1)	182 (2.1)	2493 (4.3)	588 (4.2)
Mucinous adenocarcinoma	2263 (2.8)	394 (4.7)	1323 (2.3)	546 (3.9)
Infiltrating ductal and lobular carcinoma	981 (1.2)	104 (1.2)	747 (1.3)	130 (0.9)
Infiltrating ductal and other carcinomas	1261 (1.6)	160 (1.9)	826 (1.4)	275 (2.0)
Others	4865 (6.0)	485 (5.7)	3263 (5.7)	1117 (7.9)
Biomarker expression level^a^				
HR−/HER2−	4251 (11.1)	471 (12.6)	2955 (10.8)	825 (11.4)
HR−/HER2+	4145 (10.8)	294 (7.9)	3096 (11.3)	755 (10.4)
HR+/HER2−	22,793 (59.4)	2203 (59.0)	16,097 (58.7)	4493 (62.2)
HR+/HER2+	7200 (18.7)	763 (20.5)	5284 (19.2)	1153 (16.0)
Cancer treatment				
Any treatment				
No	386 (0.5)	31 (0.4)	243 (0.4)	112 (0.8)
Yes	79,137 (99.5)	8321 (99.6)	56,976 (99.6)	13,840 (99.2)
Cancer treatment				
Treatment type				
Surgery only	3926 (5.0)	343 (4.2)	2486 (4.4)	1097 (8.0)
Surgery + neoadjuvant	2315 (3.0)	247 (3.0)	1600 (2.8)	468 (3.4)
Surgery + adjuvant	61,629 (78.5)	6405 (77.8)	44,947 (79.6)	10,277 (74.7)
Surgery + adjuvant + neoadjuvant	6326 (8.1)	928 (11.3)	4805 (8.5)	593 (4.3)
Other therapy only^b^	4267 (5.4)	308 (3.7)	2643 (4.7)	1316 (9.6)
Treatment delay (days)				
≤ 30	64,241 (81.9)	6656 (80.9)	46,249 (81.9)	11,336 (82.4)
31–60	11,062 (14.1)	1258 (15.3)	7950 (14.1)	1854 (13.5)
61–90	1547 (2.0)	155 (1.9)	1118 (2.0)	274 (2.0)
> 90	1613 (2.0)	162 (2.0)	1164 (2.0)	287 (2.1)
Body mass index (kg/m^2^)^a^				
Underweight (< 18.5)	1,699 (4.4)	395 (10.8)	1085 (3.9)	219 (3.0)
Normal (18.5−23.9)	18,361 (47.8)	2216 (60.8)	13,644 (49.5)	2501 (34.7)
Overweight (24−26.9)	9400 (24.4)	547 (15.0)	6712 (24.3)	2141 (29.7)
Slightly obese (27−29.9)	5335 (13.9)	262 (7.2)	3647 (13.2)	1426 (19.8)
Moderately obese (30−34.9)	2939 (7.6)	171 (4.7)	2010 (7.3)	758 (10.5)
Severely obese (≥ 35)	726 (1.9)	56 (1.5)	503 (1.8)	167 (2.3)
Cigarette smoking^a^				
Never	36,646 (95.3)	3349 (91.8)	26,252 (95.1)	7045 (97.7)
Former	365 (1.0)	58 (1.6)	248 (0.9)	59 (0.8)
Current	1449 (3.8)	240 (6.6)	1101 (4.0)	108 (1.5)
Alcohol drinking^a^				
Never	36,425 (94.7)	3393 (93.0)	26,039 (94.3)	6993 (97.0)
Former	193 (0.5)	17 (0.5)	149 (0.5)	27 (0.4)
Current (casual)	1419 (3.7)	183 (5.0)	1074 (3.9)	162 (2.2)
Current (habitual)	423 (1.1)	52 (1.5)	339 (1.2)	30 (0.4)

The categorical distributions for all Table 1 variables were significantly different when comparing young adult, middle-aged, and elderly patients (*p* < 0.05)

BC: Breast cancer

^a^Biomarker expression level, body mass index, cigarette smoking, and alcohol drinking were available from 2011

^b^Includes only receiving chemotherapy, radiotherapy, or hormone therapy

## Results

### Distributions of clinical and lifestyle characteristics

The distributions of clinical and lifestyle characteristics in young, middle-aged, and elderly adult breast cancer patients are shown in Table 1. Among the 80,240 female invasive breast cancer patients, 10.6% were diagnosed between 20 and 39 years of age, 71.9% were diagnosed between 40 and 64 years of age, and 17.5% were diagnosed at 65 years of age or older. Overall, most patients had more recent year at diagnosis from 2011 to 2015 (52%), stage II cancer (45.9%), infiltrating ductal carcinoma (84.3%), HR+/HER2– expression (59.4%), surgery combined with adjuvant therapy (78.5%), treatment delay ≤ 30 days (81.9%), normal BMI (48.0%), never cigarette smoking status (95.3%), and never alcohol drinking status (94.7%). After stratifying by age at diagnosis, the distributions of some variables in early-onset and later-onset breast cancer patients differed from that of the overall population. A lower proportion of young adult patients had lobular carcinoma (2.1%), HR−/HER2+ expression (7.9%), surgery only (4.2%), and BMI ≥ 24 kg/m^2^ (28.3%), whereas a higher proportion had surgery combined with adjuvant and neoadjuvant therapy (11.3%), underweight BMI (10.8%), current cigarette smoking status (6.6%), and current casual alcohol drinking status (5.0%). Among elderly patients, a higher proportion had surgery only (8.0%) and BMI ≥ 24 kg/m^2^ (62.3%), whereas a lower proportion had surgery combined with adjuvant and neoadjuvant therapy (4.3%), current cigarette smoking status (1.5%), and current alcohol drinking status (2.6%).

### Associations between age at diagnosis and 5-year survival

The associations between age at diagnosis and 5-year overall and breast cancer-specific survival in young, middle-aged, and elderly adult breast cancer patients are shown in Table 2.

**Table 2:** Association between age at diagnosis and 5-year survival of female invasive breast cancer patients

	N (%)	Five-year overall survival (%)	Adjusted RR (95% CI)	Five-year BC-specific survival (%)	Adjusted RR (95% CI)
Age at diagnosis					
Young adult	8471 (10.6)	89.9	Reference	90.5	Reference
Middle-aged	57,695 (71.9)	87.9^#^	0.99 (0.98–0.99)*	89.5	1.00 (0.99–1.00)
Elderly	14,074 (18.5)	74.5^#^	0.86 (0.85–0.87)*	83.7^#^	0.95 (0.94–0.96)*
Five-year age categories (years)					
Young adult					
20-24	114 (0.1)	82.5	Reference	83.3	Reference
25-29	725 (0.9)	88.8	1.08 (0.99–1.17)	89.7	1.08 (0.99–1.17)
30-34	2367 (3.0)	89.6	1.09 (1.00–1.18)	90.2	1.08 (1.00–1.17)
35-39	5265 (6.6)	90.3^#^	1.09 (1.01–1.18)*	90.8	1.09 (1.00–1.18)
Middle-aged					
40-44	9984 (12.4)	90.3	Reference	91.2	Reference
45-49	14,336 (17.9)	90.2	1.00 (0.99–1.01)	91.3	1.00 (1.00–1.01)
50-54	13,428 (16.7)	86.5^#^	0.97 (0.96–0.98)*	88.0^#^	0.98 (0.97–0.99)*
55-59	11,495 (14.3)	86.1^#^	0.97 (0.96–0.98)*	88.2^#^	0.98 (0.97–0.99)*
60-64	8452 (10.5)	86.0^#^	0.97 (0.96–0.98)*	88.9^#^	0.99 (0.98–1.00)
Elderly					
65-69	5624 (7.0)	83.4	Reference	87.9	Reference
70-74	3900 (4.9)	76.5^#^	0.94 (0.92–0.96)*	84.0^#^	0.97 (0.96–0.99)*
75-79	2511 (3.1)	69.1^#^	0.85 (0.83–0.88)*	81.4^#^	0.95 (0.93–0.97)*
80-84	1345 (1.7)	57.7^#^	0.73 (0.70–0.76)*	76.6^#^	0.91 (0.88–0.93)*
85+	694 (0.9)	42.1^#^	0.55 (0.51–0.60)*	69.9^#^	0.86 (0.82–0.90)*

Adjusted for year at diagnosis, cancer stage, and histological type

RR: Risk ratio, CI: Confidence interval, BC: Breast cancer

#*p* < 0.05 for unadjusted analysis; ∗*p *< 0.05 for adjusted analysis

When age at diagnosis was examined in 5-year groups, the 5-year overall survival increased from 20 to 39 years of age (82.5% for those of age 20–24 to 90.3% for those of age 35–39), and then decreased from 40 to 85 years of age or above (90.3% for those of age 40–44 to 42.1% for those of age 85 or above), whereas the 5-year breast cancer-specific survival gradually increased from 20 to 49 years of age (83.3% for those of age 20–24 to 91.3% for those of age 45–49), declined and then increased from 50 to 64 years of age (88.0% for those of age 50–54 to 88.9% for those of age 60–64), and decreased from age 60 to 85 years or above (88.9% for those of age 60–64 to 69.9% for those of age over 85). Supplemental Fig. 1 illustrates the 5-year survival rates by age at diagnosis using line graphs. We obtained similar findings after adjusting for year at diagnosis, cancer stage, and histological type, but those diagnosed at 60–64 years of age no longer had significantly worse survival than those diagnosed at 40–44 years of age.

### Associations between clinical characteristics and 5-year survival

Table 3 shows the 5-year overall survival in young, middle-aged, and elderly breast cancer patients by clinical characteristics, as well as the adjusted RR for the associations with overall survival. Compared with infiltrating ductal carcinoma patients, mucinous adenocarcinoma patients had significantly better survival, whereas patients with other histological types had significantly worse survival. After stratifying by age at diagnosis, the results in the three age groups were mostly similar, but compared with infiltrating ductal carcinoma patients, elderly patients with infiltrating duct mixed with other carcinoma had significantly better survival. For biomarker expression level, patients with HR+/HER2− had the highest survival rate. Compared with HR−/HER2- expression patients, those with other combinations of HR and HER2 had significantly better survival. After stratifying by age at diagnosis, the results in the three age groups were mostly similar, except for the statistically insignificant results in elderly patients with HR−/HER2+ expression. For the analysis involving receiving any treatment, treated patients had significantly better survival in the three age groups, but there was more noticeable improvement in elderly patients. For treatment type, the results in the three age groups were mostly similar, but receiving surgery plus neoadjuvant therapy improved overall survival only in elderly patients compared with receiving only surgery, whereas receiving surgery plus adjuvant therapy improved survival in all three groups. Although those with treatment delay had worse 5-year survival, treatment delay did not adversely influence survival after adjustment for confounding. The crude RR and 95% CI for overall survival are presented in Supplemental Table 1. The 5-year breast cancer-specific survival findings were similar (Supplemental Table 2).

**Table 3:** Associations between clinical characteristics and 5-year overall survival of female invasive breast cancer patients

Clinical Characteristics	All BC		Young adult BC		Middle-aged BC		Elderly BC	
	Five-year survival (%)	Adjusted RR (95% CI)	Five-year survival (%)	Adjusted RR (95% CI)	Five-year survival (%)	Adjusted RR (95% CI)	Five-year survival (%)	Adjusted RR (95% CI)
Cancer stage								
I	96.2	Reference	97.4	Reference	97.3	Reference	89.9	Reference
II	89.0^#^	0.93 (0.93–0.93)*	92.4^#^	0.95 (0.94–0.96)*	91.1^#^	0.94 (0.93–0.94)*	78.7^#^	0.90 (0.89–0.92)*
III	67.9^#^	0.72 (0.70–0.73)*	73.6^#^	0.76 (0.73–0.79)*	71.6^#^	0.74 (0.73–0.75)*	53.7^#^	0.63 (0.61–0.66)*
IV	28.3^#^	0.30 (0.29–0.31)*	36.4^#^	0.37 (0.33–0.43)*	29.8^#^	0.31 (0.29–0.32)*	20.9^#^	0.24 (0.22–0.27)*
Histological type								
Infiltrating ductal carcinoma	85.8	Reference	89.4	Reference	87.9	Reference	74.5	Reference
Lobular carcinoma	85.4	1.01 (1.00–1.02)	89.0	1.00 (0.96–1.04)	88.1	1.01 (1.00–1.02)	73.0	0.99 (0.95–1.04)
Mucinous adenocarcinoma	93.7^#^	1.05 (1.04–1.06)*	98.5^#^	1.06 (1.05–1.08)*	97.1^#^	1.06 (1.05–1.07)*	82.1^#^	1.10 (1.06–1.14)*
Infiltrating ductal and lobular carcinoma	88.3^#^	1.01 (0.99–1.03)	90.4	1.02 (0.96–1.08)	90.0	1.00 (0.98–1.02)	76.9	1.00 (0.92–1.08)
Infiltrating ductal and other carcinomas	89.3^#^	1.02 (1.00–1.04)	92.5	1.02 (0.98–1.06)	91.3^#^	1.02 (1.00–1.04)	81.5^#^	1.07 (1.01–1.13)*
Others	80.5^#^	0.98 (0.96–0.99)*	88.5	1.00 (0.97–1.03)	83.3^#^	0.97 (0.96–0.99)*	69.1^#^	0.99 (0.96–1.03)
Biomarker expression level^a^								
HR−/HER2−	78.5	Reference	81.3	Reference	81.2	Reference	67.4	Reference
HR−/HER2+	80.9^#^	1.07 (1.05–1.09)*	85.0	1.08 (1.03–1.15)*	83.8^#^	1.06 (1.04–1.08)*	67.5	1.03 (0.97–1.10)
HR+/HER2−	89.5^#^	1.11 (1.09–1.13)*	93.4^#^	1.13 (1.08–1.17)*	91.6^#^	1.10 (1.09–1.12)*	80.2^#^	1.13 (1.09–1.18)*
HR+/HER2+	86.0^#^	1.11 (1.09–1.13)*	90.7^#^	1.14 (1.09–1.19)*	87.9^#^	1.10 (1.08–1.12)*	74.2^#^	1.12 (1.06–1.18)*
Cancer treatment								
Any treatment								
No	45.1	Reference	77.4	Reference	49.4	Reference	26.8	Reference
Yes	86.1^#^	1.55 (1.41–1.70)*	90.0	1.11 (0.95–1.30)	88.2^#^	1.44 (1.30–1.60)*	75.2^#^	2.10 (1.57–2.80)*
Treatment type								
Surgery only	82.9	Reference	89.2	Reference	88.9	Reference	67.5	Reference
Surgery + neoadjuvant	63.1^#^	0.95 (0.92–0.98)*	66.0^#^	0.88 (0.80–0.96)*	63.4^#^	0.90 (0.87–0.94)*	60.7^#^	1.09 (1.01–1.17)*
Surgery + adjuvant	91.6^#^	1.08 (1.07–1.10)*	94.2^#^	1.05 (1.01–1.09)*	93.3^#^	1.04 (1.03–1.06)*	82.9^#^	1.16 (1.12–1.21)*
Surgery + adjuvant + neoadjuvant	79.1^#^	1.09 (1.07–1.11)*	83.1^#^	1.04 (0.99–1.09)	79.4^#^	1.04 (1.02–1.06)*	71.2	1.24 (1.16–1.32)*
Other therapy only^b^	30.2^#^	0.62 (0.59–0.65)*	43.2^#^	0.73 (0.64–0.83)*	30.6^#^	0.61 (0.58–0.65)*	26.5^#^	0.66 (0.60–0.73)*
Treatment delay (days)								
≤ 30	86.2	Reference	90.3	Reference	88.2	Reference	75.4	Reference
31–60	87.1	1.01 (1.01–1.02)*	90.0	1.00 (0.99–1.02)	89.4#	1.02 (1.01–1.02)*	75.1	0.99 (0.97–1.02)
61–90	83.7#	1.00 (0.98–1.02)	85.2	0.98 (0.93–1.04)	86.0	1.01 (0.99–1.03)	73.4	1.00 (0.94–1.06)
> 90	74.8#	1.00 (0.98–1.03)	78.4#	0.98 (0.91–1.06)	76.9#	1.00 (0.97–1.02)	64.1#	1.02 (0.95–1.10)

All adjusted analyses adjusted for year at diagnosis and age at diagnosis. For the adjusted analyses involving cancer stage and histological type, they were mutually adjusted. For the adjusted analyses involving biomarker expression level and any cancer treatment, they were adjusted for cancer stage and histological type. For the adjusted analyses involving treatment type and treatment delay, they were mutually adjusted and adjusted for cancer stage and histological type

RR: Risk ratio, CI: Confidence interval, BC: Breast cancer

#*p* < 0.05 for unadjusted analysis; ∗*p *< 0.05 for adjusted analysis

^a^Biomarker expression level was available from 2011

^b^Includes only receiving chemotherapy, radiotherapy, or hormone therapy

### Associations between lifestyle characteristics and 5-year survival

Table 4 shows the 5-year overall survival in young, middle-aged, and elderly breast cancer patients by lifestyle characteristics, as well as the adjusted RR for the associations with overall survival. Compared with patients with normal BMI, underweight and severely obese patients had significantly worse survival in the overall population. After stratifying by age at diagnosis, severely obese patients had worse survival in young and middle-aged adult patients, which reached statistical significance for middle-aged patients, while underweight patients had significantly worse survival in middle-aged and especially in elderly patients. For cigarette smoking, current smokers had worse survival than never smokers in middle-aged and elderly patients, which reached statistical significance for middle-aged patients. For alcohol drinking, there were no significant associations in the adjusted analysis. The crude RR and 95% CI for overall survival are presented in Supplemental Table 3. The 5-year breast cancer-specific survival results were similar (Supplemental Table 4).

**Table 4:** Associations between lifestyle characteristics and 5-year overall survival of female invasive breast cancer patients

Lifestyle Characteristics	All BC		Young adult BC		Middle-aged BC		Elderly BC	
	Five-year survival (%)	Adjusted RR (95% CI)	Five-year survival (%)	Adjusted RR (95% CI)	Five-year survival (%)	Adjusted RR (95% CI)	Five-year survival (%)	Adjusted RR (95% CI)
Body mass index (kg/m^2^ )								
Normal (18.5–23.9)	87.8	Reference	91.0	Reference	89.6	Reference	75.4	Reference
Underweight (< 18.5)	79.8^#^	0.94 (0.92–0.96)*	90.9	1.00 (0.97–1.03)	80.7^#^	0.96 (0.94–0.98)*	54.8^#^	0.82 (0.74–0.91)*
Overweight (24–26.9)	87.5	1.02 (1.01–1.02)*	90.1	1.00 (0.97–1.03)	89.6	1.01 (1.00–1.02)	80.1^#^	1.03 (1.00–1.06)
Slightly obese (27–29.9)	87.1	1.02 (1.01–1.03)*	87.4	0.98 (0.93–1.02)	89.2	1.01 (1.00–1.02)	81.7^#^	1.05 (1.02–1.09)*
Moderately obese (30–34.9)	85.9^#^	1.01 (0.99–1.02)	94.7	1.03 (0.99–1.08)	87.7^#^	0.99 (0.98–1.01)	79.3	1.03 (0.99–1.07)
Severely obese (≥ 35)	81.7^#^	0.96 (0.93–0.99)*	82.1	0.94 (0.84–1.05)	83.9^#^	0.94 (0.91–0.98)*	74.9	0.99 (0.91–1.07)
Cigarette smoking								
Never	87.1	Reference	90.6	Reference	89.1	Reference	78.0	Reference
Former	85.8	1.00 (0.96–1.03)	89.7	0.99 (0.93–1.07)	88.3	1.02 (0.98–1.06)	71.2	0.94 (0.82–1.09)
Current	85.9	0.96 (0.95–0.98)^*^	90.8	0.98 (0.94–1.02)	86.5^#^	0.97 (0.95–0.99)*	68.5	0.90 (0.80–1.02)
Alcohol drinking								
Never	87.0	Reference	90.6	Reference	89.0	Reference	77.6	Reference
Former	80.8	0.99 (0.93–1.05)	88.2	1.05 (0.95–1.15)	80.5^#^	0.96 (0.90–1.03)	77.8	1.06 (0.90–1.25)
Current (casual)	89.6^#^	1.01 (0.99–1.03)	92.3	1.02 (0.98–1.07)	89.7	1.00 (0.98–1.02)	85.8^#^	1.06 (1.00–1.12)
Current (habitual)	87.5	1.01 (0.97–1.04)	85.2	0.95 (0.86–1.06)	88.8	1.01 (0.97–1.04)	76.7	1.04 (0.88–1.24)

For the adjusted analysis involving body mass index, cigarette smoking, and alcohol drinking, they were mutually adjusted and adjusted for year at diagnosis, age at diagnosis, cancer stage, and histological type

RR: Risk ratio, CI: Confidence interval, BC: Breast cancer

#*p* < 0.05 for unadjusted analysis; ∗*p *< 0.05 for adjusted analysis

### Causes of death

The distributions of the causes of death stratified by age at diagnosis are shown in Table 5. Overall, most patients died from neoplastic causes involving breast cancer (80.1%) or other cancers (6.1%). Regardless of age at diagnosis, the leading causes of death were breast cancer or other cancer (young adults: 94.1 and 3.2%, respectively; middle-aged: 86.7 and 5.6%, respectively; elderly: 63.9 and 7.7%, respectively), but among young and middle-aged adult patients, the next leading cause of death was suicide (1.1% and 1.5%, respectively), whereas among elderly patients, the next leading causes of death were circulatory system diseases (10.1%), respiratory system diseases (4.0%), and endocrine, nutritional and metabolic diseases (3.3%).

**Table 5:** Causes of death among young, middle-aged, and elderly adult female invasive breast cancer patients

Characteristics	All BC (N, %)	Young adult BC (N, %)	Middle-aged BC (N, %)	Elderly BC (N, %)
Cause of death				
Certain infectious and parasitic diseases	129 (1.1)	3 (0.35)	54 (0.77)	72 (2.0)
Neoplasms (Breast cancer)	9150 (80.1)	807 (94.1)	6045 (86.7)	2298 (63.9)
Neoplasms (Non-breast cancer)	692 (6.1)	27 (3.2)	389 (5.6)	276 (7.7)
Diseases of the blood and blood-forming organs and certain disorders involving the immune mechanism	12 (0.11)	0	7 (0.10)	5 (0.14)
Endocrine, nutritional and metabolic diseases	161 (1.4)	0	43 (0.62)	118 (3.3)
Mental and behavioral disorders	12 (0.11)	0	2 (0.03)	10 (0.28)
Diseases of the nervous system	24 (0.21)	0	9 (0.13)	15 (0.42)
Diseases of the circulatory system	457 (4.0)	3 (0.35)	91 (1.3)	363 (10.1)
Diseases of the respiratory system	195 (1.7)	1 (0.12)	49 (0.70)	145 (4.0)
Diseases of the digestive system	152 (1.3)	2 (0.23)	69 (0.99)	81 (2.3)
Diseases of the skin and subcutaneous tissue	9 (0.08)	0	5 (0.07)	4 (0.11)
Diseases of the musculoskeletal system and connective tissue	20 (0.18)	1 (0.12)	3 (0.04)	16 (0.45)
Diseases of the genitourinary system	140 (1.2)	0	38 (0.55)	102 (2.8)
Symptoms, signs and abnormal clinical and laboratory findings, not elsewhere classified	43 (0.38)	1 (0.12)	14 (0.20)	28 (0.78)
External causes of morbidity and mortality (excluding suicide)	90 (0.79)	4 (0.47)	48 (0.68)	38 (1.1)
Suicide	138 (1.2)	9 (1.1)	105 (1.5)	24 (0.67)

BC: Breast cancer

## Discussion

To our knowledge, this is the largest study to date on the 5-year survival of young, middle-aged, and elderly female adult invasive breast cancer patients according to clinical and lifestyle characteristics. We found that compared with the overall and older breast cancer population, a lower proportion of young adult patients had stage IV cancer, lobular carcinoma, HR−/HER2+ expression, only surgery treatment, and BMI ≥ 24 kg/m^2^, whereas a higher proportion had surgery combined with adjuvant and neoadjuvant therapy, normal and underweight BMI, current cigarette smoking status, and current casual alcohol drinking status. The 5-year breast cancer-specific survival was not significantly better for those diagnosed at age 45–49 years than at 40–44 years, despite the recommended starting screening age is 45 years in Taiwan. Among young adult breast cancer patients, survival was the worst in those 20–24 years of age and improved with increasing age. Receiving any treatment was associated with better survival for middle-aged patients and especially for elderly ones. Compared with receiving only surgery, receiving surgery and neoadjuvant therapy improved overall survival only in elderly patients, whereas receiving surgery and adjuvant therapy improved survival for young, middle-aged, and elderly adult patients. Current smokers had worse survival than never smokers for middle-aged and elderly patients, which reached statistical significance for middle-aged patients. Being underweight at initial cancer treatment was associated with significantly worse survival than having normal weight, for middle-aged and especially for elderly patients.

### Age at diagnosis

Our findings for the association between age at diagnosis and 5-year survival were supported by a Korean study showing that patients younger than 40 years and older than 60 years of age had worse survival than patients 40–49 years of age [14]. Due to lack of awareness and early examinations, delayed diagnosis often occurs in younger women [15, 16]. Young adult breast cancer patients were found to be diagnosed at more advanced stages than older patients [17, 18], and those diagnosed at 15–29 years of age had more advanced diseases than those diagnosed at 30–39 years of age [18]. Although we did not find a higher proportion of advanced-stage cancers in young adult patients compared with middle-aged or elderly patients, young adult breast cancers had a higher proportion of aggressive molecular subtypes such as triple-negative breast cancer, which may explain the worse survival in young adult patients. Additional studies are needed to evaluate the cost-effectiveness of lowering the starting age for breast cancer screening in Taiwan to 40 years.

### Tumor characteristics

Previous studies have indicated that compared with infiltrating ductal carcinoma patients, mucinous adenocarcinoma patients had better prognosis, which is consistent with our findings for all three age at diagnosis groups [19–22]. Our findings on biomarker expression level indicate that patients with HR− expression have worse survival than those with HR+ expression, especially those with additional HER2− expression, which is similar to results of previous studies [7, 23, 24]. A previous study showed that among elderly women diagnosed at 70–89 years of age, those with HR−/HER2+ expression had significantly worse survival compared with those with HR+/HER2− expression [7], whereas we found this association across all three age at diagnosis groups.

### Treatment characteristics

In our study, we found a higher proportion of elderly patients received only surgery, and a higher proportion of young adult patients received surgery combined with adjuvant and neoadjuvant therapy, which were consistent with previous studies [25, 26]. Moreover, we found a higher proportion of elderly patients received only chemotherapy, hormone therapy, or radiotherapy, which was also consistent with previous studies [27, 28]. In this study, there was a slightly lower proportion of elderly patients receiving any treatment than younger patients. However, we found elderly patients did not more often receive delayed treatment, which was contrary to a European study that found elderly patients received treatment less promptly [28]. We found receiving treatment improved survival more for elderly patients, which supports providing them with standard treatments [28, 29]. Furthermore, we found those who received surgery had better survival than those who did not, which was consistent with previous studies [26, 30, 31].

Compared with patients who received only surgery, we found receiving surgery with adjuvant therapy improved survival in all three age groups, whereas there was significantly reduced survival among young adult and middle-aged patients who received surgery with neoadjuvant therapy, after adjusting for confounding factors. We found significantly improved overall survival among elderly patients who received surgery with neoadjuvant therapy compared with those receiving only surgery, but there was no significant association involving breast-cancer specific survival. This suggests that elderly patients receiving surgery with neoadjuvant therapy in our study might be healthier than those who received only surgery. On the other hand, younger patients in our study who received neoadjuvant chemotherapy and surgery were likely those with more aggressive types of breast cancer and thus had poorer prognosis. A previous study showed that neoadjuvant systemic therapy in carefully-selected elderly patients, such as those with low scores for comorbidity and toxicity risk from chemotherapy, is an appropriate treatment modality to improve overall survival [32]. The role of neoadjuvant therapy on breast cancer survival in young, middle-aged, and elderly breast cancer patients should be further evaluated in future studies.

Previous studies have indicated no association between breast cancer treatment delay and survival [33–35], which was similar to our confounding-adjusted findings. A previous meta-analysis suggested that treatment delay was associated with increased risk of mortality for surgery, adjuvant systemic treatment, and neoadjuvant systemic treatment in breast cancer patients [36].

### Lifestyle characteristics

We found young adult patients had higher proportion of underweight BMI compared with older adult patients, whereas there was a higher proportion of elderly patients with overweight and obese BMI compared with younger patients, which was consistent with some previous studies [8, 37–39]. Compared with patients having normal BMI, having underweight or severely obese BMI in our study was associated with significantly worse survival for the overall population after confounding adjustment. After stratifying by age at diagnosis, similar results for underweight BMI were found among middle-aged and elderly patients, but not in young adult patients. The association between being underweight and poorer breast cancer prognosis has been found by previous studies [39–41]. Underweight BMI due to weight loss from other comorbidities may especially adversely affect the survival of older patients. Previous studies had similar findings among patients over the age of 70 years [8] and young patients 18 to 40 years of age [38]. In terms of being overweight, previous studies indicated significantly worse survival among patients with overweight or obese BMI [37, 40, 42, 43], whereas another study demonstrated no association of overweight and obesity with worse prognosis among metastatic breast cancer patients [41]. For patients in our overall population who were overweight or heavier (BMI ≥ 24 kg/m^2^), only severe obesity adversely influenced overall survival after confounding adjustment. We found elderly patients with slightly obese BMI had better survival compared with having normal BMI, whereas young adult and middle-aged patients with severely obese BMI had worse survival, but this did not reach statistical significance for young adult patients. A previous study indicated higher BMI was associated with higher mortality among patients over the age of 70 years, but this result did not achieve statistical significance [8].

We found higher proportions of young adult patients who were current smokers or drinkers, compared with older patients, similar to previous studies on alcohol consumption [44, 45] and cigarette smoking [46, 47] in breast cancer patients. We found there was no significant association between alcohol drinking and survival, whereas previous studies showed alcohol consumption was associated with increased risk of breast cancer-specific mortality [44, 45]. In addition, we found that compared with never smokers, current smokers had significantly worse survival among middle-aged and elderly patients, which was supported by previous studies that demonstrated smoking was associated with increased breast cancer mortality risk [9, 46, 47].

### Strengths and limitations

To our knowledge, this is the first study to comprehensively examine the 5-year survival of young, middle-aged, and elderly female adult invasive breast cancer patients by clinical and lifestyle characteristics. We used high-quality nationwide data from the Taiwan Cancer Registry, which helped reduce information bias and enhance generalizability to the Taiwanese population [48]. Furthermore, the Taiwan Cancer Registry contains information on BMI, cigarette smoking, and alcohol drinking, which are usually not collected in other cancer registries. This study also has some limitations. Although we adjusted for confounding to help explain our findings, we did not have information on potential unmeasured confounders such as socioeconomic factors, reproductive factors, and physical activity. Furthermore, information on lifestyle factors and biomarker expression level were only available from 2011 and afterwards, which reduced the sample sizes for analyses involving those variables.

## Conclusion

Our findings on the 5-year survival of female breast cancer patients by age at diagnosis contribute to the understanding of early-onset and later-onset breast cancer characteristics and prognosis, and may inform surveillance and treatment strategies to achieve better breast cancer prognosis. Future studies are needed to better understand the association of these characteristics with 5-year survival in other populations of early-onset and later-onset breast cancer patients. Furthermore, future studies can investigate how detailed treatment characteristics, such as surgery types, radiation dose, and specific chemotherapy drugs are associated with survival prognosis in early-onset and later-onset breast cancer patients.

### Supplementary Information

Below is the link to the electronic supplementary material.

Supplementary material 1 (DOCX 82.8 kb)

## Data Availability

No datasets were generated or analysed during the current study.
