# Correction: Five-year survival prognosis of young, middle-aged, and elderly adult female invasive breast cancer patients by clinical and lifestyle characteristics

**DOI:** 10.1007/s10549-024-07335-5

**Published:** 2024-05-02

**Authors:** Yu-Tung Teng, Yong Alison Wang, Yaa-Hui Dong, Jason J. Liu

**Affiliations:** 1 Institute of Public Health, National Yang Ming Chiao Tung University, No.155, Sec. 2, Linong St., Beitou District, Taipei, 112 Taiwan; 2 Koo Foundation Sun-Yat Sen Cancer Center, Taipei, Taiwan; 3 School of Medicine, National Yang Ming Chiao Tung University, Taipei, Taiwan; 4 Department of Pharmacy, National Yang Ming Chiao Tung University, Taipei, Taiwan

**Breast Cancer Research and Treatment**

10.1007/s10549-024-07280-3

In Figure 1 of this article, the following errors have been made during the production process.

**Fig. 1:**
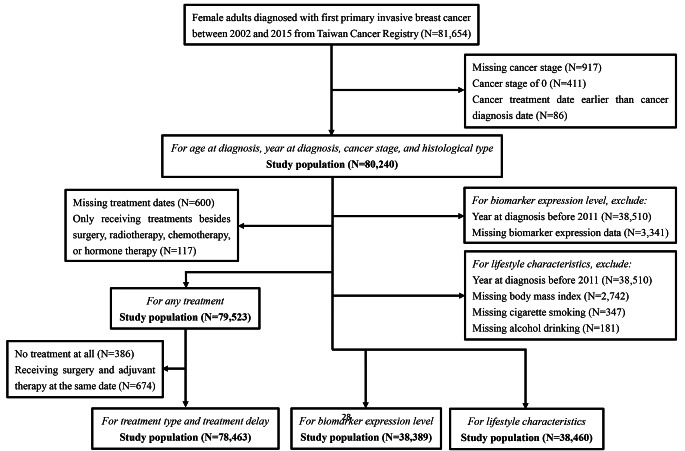
Flow chart for the selection of the study population

1. Sample size N = 117 for "Only receiving treatments besides surgery, radiotherapy, chemotherapy, or hormone therapy" was missed out, and should have been Only receiving treatments besides surgery, radiotherapy, chemotherapy, or hormone therapy (N = 117)"

2. The spacing between surgery and radiotherapy is too large

3. There should be no comma between treatment type and treatment delay, and should have been "For treatment type and treatment delay". The Fig. 1 should have appeared as shown below.

The original article has been corrected.

