# Supplementary material for: Five-year survival prognosis of young, middle-aged, and elderly adult female invasive breast cancer patients by clinical and lifestyle characteristics
Source: Breast Cancer Res Treat. 2024 Mar 25;205(3):619–31. doi: 10.1007/s10549-024-07280-3 (PMC11101574; doi:10.1007/s10549-024-07280-3)

**Supplemental Table 1.** **Crude risk ratios for the unadjusted association between clinical characteristics and five-year overall survival**

| Clinical Characteristics | All BC | | Young adult BC | | | Middle-aged BC | | | Elderly BC | | |  | |  |
| --- | --- | --- | --- | --- | --- | --- | --- | --- | --- | --- | --- | --- | --- | --- |
|  | Five-year survival (%) | Crude RR  (95% CI) | | Five-year survival (%) | Crude RR  (95% CI) | | Five-year survival (%) | Crude RR  (95% CI) | | Five-year survival (%) |  | | Crude RR  (95% CI) | |
| **Cancer stage** |  |  | |  |  | |  |  | |  |  | |  | |
| Ⅰ | 96.2 | Reference | | 97.4 | Reference | | 97.3 | Reference | | 89.9 |  | | Reference | |
| Ⅱ | 89.0 | 0.92 (0.92-0.93)* | | 92.4 | 0.95 (0.94-0.96)* | | 91.1 | 0.94 (0.93-0.94)* | | 78.7 |  | | 0.88 (0.86-0.89)* | |
| Ⅲ | 67.9 | 0.71 (0.70-0.72)* | | 73.6 | 0.76 (0.73-0.79)* | | 71.6 | 0.74 (0.73-0.75)* | | 53.7 |  | | 0.60 (0.57-0.62)* | |
| Ⅳ | 28.3 | 0.29 (0.28-0.31)* | | 36.4 | 0.37 (0.33-0.43)* | | 29.8 | 0.31 (0.29-0.32)* | | 20.9 |  | | 0.23 (0.21-0.26)* | |
| **Histological type** |  |  | |  |  | |  |  | |  |  | |  | |
| Infiltrating ductal carcinoma | 85.8 | Reference | | 89.4 | Reference | | 87.9 | Reference | | 74.5 |  | | Reference | |
| Lobular carcinoma | 85.4 | 1.00 (0.98-1.01) | | 89.0 | 1.00 (0.94-1.05) | | 88.1 | 1.00 (0.99-1.02) | | 73.0 |  | | 0.98 (0.93-1.03) | |
| Mucinous adenocarcinoma | 93.7 | 1.09 (1.08-1.10)* | | 98.5 | 1.10 (1.08-1.12)* | | 97.1 | 1.11 (1.09-1.12)* | | 82.1 |  | | 1.10 (1.06-1.15)* | |
| Infiltrating ductal and lobular carcinoma | 88.3 | 1.03 (1.01-1.05) | | 90.4 | 1.01 (0.95-1.08) | | 90.0 | 1.02 (1.00-1.05) | | 76.9 |  | | 1.03 (0.94-1.14) | |
| Infiltrating ductal and other carcinomas | 89.3 | 1.04 (1.02-1.06) | | 92.5 | 1.03 (0.99-1.08) | | 91.3 | 1.04 (1.02-1.06)* | | 81.5 |  | | 1.09 (1.03-1.16)* | |
| Others | 80.5 | 0.94 (0.93-0.95)* | | 88.5 | 0.99 (0.96-1.02) | | 83.3 | 0.95 (0.93-0.96)* | | 69.1 |  | | 0.93 (0.89-0.97)* | |
| **Biomarker expression level^a^** |  |  | |  |  | |  |  | |  |  | |  | |
| HR-/HER2- | 78.5 | Reference | | 81.3 | Reference | | 81.2 | Reference | | 67.4 |  | | Reference | |
| HR-/HER2+ | 80.9 | 1.03 (1.01-1.05)* | | 85.0 | 1.05 (0.98-1.12) | | 83.8 | 1.03 (1.01-1.06)* | | 67.5 |  | | 1.00 (0.94-1.07) | |
| HR+/HER2- | 89.5 | 1.14 (1.12-1.16)* | | 93.4 | 1.15 (1.10-1.20)* | | 91.6 | 1.13 (1.11-1.15)* | | 80.2 |  | | 1.19 (1.13-1.25)* | |
| HR+/HER2+ | 86.0 | 1.09 (1.07-1.11)* | | 90.7 | 1.12 (1.06-1.17)* | | 87.9 | 1.08 (1.06-1.10)* | | 74.2 |  | | 1.10 (1.04-1.17)* | |

| Clinical Characteristics | All BC | | Young adult BC | | | Middle-aged BC | | | Elderly BC | | |  |
| --- | --- | --- | --- | --- | --- | --- | --- | --- | --- | --- | --- | --- |
|  | Five-year survival (%) | Crude RR  (95% CI) | | Five-year survival (%) | Crude RR  (95% CI) | | Five-year survival (%) | Crude RR  (95% CI) | | Five-year survival (%) | Crude RR  (95% CI) | |
| **Cancer treatment** |  |  | |  |  | |  |  | |  |  | |
| Any treatment |  |  | |  |  | |  |  | |  |  | |
| No | 45.1 | Reference | | 77.4 | Reference | | 49.4 | Reference | | 26.8 | Reference | |
| Yes | 86.1 | 1.91 (1.71-2.13)* | | 90.0 | 1.16 (0.96-1.41) | | 88.2 | 1.79 (1.57-2.03)* | | 75.2 | 2.81 (2.07-3.81)* | |
| Treatment type |  |  | |  |  | |  |  | |  |  | |
| Surgery only | 82.9 | Reference | | 89.2 | Reference | | 88.9 | Reference | | 67.5 | Reference | |
| Surgery+neoadjuvant | 63.1 | 0.76 (0.74-0.79)* | | 66.0 | 0.74 (0.67-0.81)* | | 63.4 | 0.71 (0.69-0.74)* | | 60.7 | 0.90 (0.83-0.98)* | |
| Surgery+adjuvant | 91.6 | 1.11 (1.09-1.12)* | | 94.2 | 1.06 (1.02-1.10)* | | 93.3 | 1.05 (1.03-1.06)* | | 82.9 | 1.23 (1.18-1.28)* | |
| Surgery+adjuvant+  neoadjuvant | 79.1 | 0.95 (0.94-0.97)* | | 83.1 | 0.93 (0.89-0.98)* | | 79.4 | 0.89 (0.88-0.91)* | | 71.2 | 1.05 (0.99-1.13) | |
| Other therapy only^b^ | 30.2 | 0.36 (0.35-0.38)* | | 43.2 | 0.48 (0.42-0.55)* | | 30.6 | 0.34 (0.32-0.36)* | | 26.5 | 0.39 (0.36-0.43)* | |
| **Treatment delay (days)** |  |  | |  |  | |  |  | |  |  | |
| ≤30 | 86.2 | Reference | | 90.3 | Reference | | 88.2 | Reference | | 75.4 | Reference | |
| 31-60 | 87.1 | 1.01 (1.00-1.02) | | 90.0 | 1.00 (0.98-1.02) | | 89.4 | 1.01 (1.01-1.02)* | | 75.1 | 1.00 (0.97-1.02) | |
| 61-90 | 83.7 | 0.97 (0.95-0.99)* | | 85.2 | 0.94 (0.88-1.01) | | 86.0 | 0.98 (0.95-1.00) | | 73.4 | 0.97 (0.91-1.05) | |
| >90 | 74.8 | 0.87 (0.84-0.89)* | | 78.4 | 0.87 (0.80-0.94)* | | 76.9 | 0.87 (0.84-0.90)* | | 64.1 | 0.85 (0.78-0.93)* | |

RR: risk ratio; CI: confidence interval; BC: breast cancer. **p*<0.05

^a^Biomarker expression level was available from 2011.

^b^Includes only receiving chemotherapy, radiotherapy, or hormone therapy.

**Supplemental Table 2. Associations between clinical characteristics and five-year breast cancer-specific survival of female invasive breast cancer patients**

| Clinical Characteristics | All BC | | Young adult BC | | | Middle-aged BC | | | Elderly BC | | |  |
| --- | --- | --- | --- | --- | --- | --- | --- | --- | --- | --- | --- | --- |
|  | Five-year survival (%) | Adjusted RR  (95% CI)^b^ | | Five-year survival (%) | Adjusted RR  (95% CI)^b^ | | Five-year survival (%) | Adjusted RR  (95% CI)^b^ | | Five-year survival (%) | Adjusted RR  (95% CI)^b^ | |
| **Cancer stage** |  |  | |  |  | |  |  | |  |  | |
| Ⅰ | 98.0 | Reference | | 97.8 | Reference | | 98.4 | Reference | | 96.4 | Reference | |
| Ⅱ | 92.0^#^ | 0.94 (0.94-0.94)* | | 92.9^#^ | 0.95 (0.94-0.96)* | | 92.7^#^ | 0.94 (0.94-0.95)* | | 88.6^#^ | 0.93 (0.92-0.94)* | |
| Ⅲ | 72.8^#^ | 0.75 (0.74-0.76)* | | 74.6^#^ | 0.76 (0.73-0.79)* | | 74.4^#^ | 0.76 (0.75-0.77)* | | 66.9^#^ | 0.71 (0.68-0.73)* | |
| Ⅳ | 33.1^#^ | 0.34 (0.33-0.35)* | | 38.8^#^ | 0.40 (0.33-0.43)* | | 33.3^#^ | 0.34 (0.32-0.36)* | | 30.5^#^ | 0.32 (0.29-0.35)* | |
| **Histological type** |  |  | |  |  | |  |  | |  |  | |
| Infiltrating ductal carcinoma | 88.5 | Reference | | 90.1 | Reference | | 89.4 | Reference | | 83.5 | Reference | |
| Lobular carcinoma | 88.0 | 1.01 (0.99-1.02) | | 90.1 | 1.00 (0.96-1.04) | | 89.5 | 1.01 (0.99-1.02) | | 81.3 | 0.99 (0.96-1.02) | |
| Mucinous adenocarcinoma | 97.4^#^ | 1.06 (1.05-1.07)* | | 98.5^#^ | 1.06 (1.05-1.08)* | | 98.4^#^ | 1.06 (1.05-1.07)* | | 94.1^#^ | 1.10 (1.07-1.12)* | |
| Infiltrating ductal and lobular carcinoma | 89.8 | 1.00 (0.98-1.02) | | 91.3 | 1.02 (0.96-1.08) | | 91.2 | 1.00 (0.98-1.02) | | 80.8 | 0.95 (0.89-1.02) | |
| Infiltrating ductal and other carcinomas | 92.5^#^ | 1.02 (1.01-1.04) | | 93.8 | 1.02 (0.98-1.06) | | 93.1^#^ | 1.02 (1.00-1.04) | | 90.2^#^ | 1.04 (1.01-1.08)* | |
| Others | 84.9^#^ | 0.99 (0.98-1.00) | | 89.1 | 1.00 (0.97-1.03) | | 85.8^#^ | 0.98 (0.97-1.00) | | 80.4^#^ | 1.01 (0.99-1.04) | |
| **Biomarker expression level^a^** |  |  | |  |  | |  |  | |  |  | |
| HR-/HER2- | 81.7 | Reference | | 82.2 | Reference | | 83.1 | Reference | | 76.6 | Reference | |
| HR-/HER2+ | 83.2^#^ | 1.05 (1.03-1.07)* | | 85.0 | 1.07 (1.01-1.13)* | | 84.9 | 1.05 (1.03-1.07)* | | 75.6 | 1.02 (0.97-1.07) | |
| HR+/HER2- | 92.3^#^ | 1.11 (1.09-1.12)* | | 94.0^#^ | 1.12 (1.08-1.17)* | | 93.0^#^ | 1.10 (1.08-1.11)* | | 89.0^#^ | 1.12 (1.08-1.16)* | |
| HR+/HER2+ | 88.6^#^ | 1.10 (1.09-1.12)* | | 91.0^#^ | 1.13 (1.08-1.18)* | | 89.6^#^ | 1.10 (1.08-1.12)* | | 82.7^#^ | 1.10 (1.06-1.15)* | |

| Clinical Characteristics | All BC | | Young adult BC | | | Middle-aged BC | | | Elderly BC | | |  |
| --- | --- | --- | --- | --- | --- | --- | --- | --- | --- | --- | --- | --- |
|  | Five-year survival (%) | Adjusted RR  (95% CI)^b^ | | Five-year survival (%) | Adjusted RR  (95% CI)^b^ | | Five-year survival (%) | Adjusted RR  (95% CI)^b^ | | Five-year survival (%) | Adjusted RR  (95% CI)^b^ | |
| **Cancer treatment** |  |  | |  |  | |  |  | |  |  | |
| Any treatment |  |  | |  |  | |  |  | |  |  | |
| No | 56.0 | Reference | | 77.4 | Reference | | 57.2 | Reference | | 47.3 | Reference | |
| Yes | 88.9^#^ | 1.32 (1.22-1.43)* | | 90.6 | 1.12 (0.96-1.31) | | 89.8^#^ | 1.28 (1.17-1.41)* | | 84.3^#^ | 1.47 (1.22-1.77)* | |
| Treatment type |  |  | |  |  | |  |  | |  |  | |
| Surgery only | 89.0 | Reference | | 90.1 | Reference | | 91.6 | Reference | | 82.8 | Reference | |
| Surgery+neoadjuvant | 67.2^#^ | 0.93 (0.90-0.96)* | | 67.2^#^ | 0.88 (0.80-0.96)* | | 66.3^#^ | 0.90 (0.87-0.93)* | | 70.1^#^ | 1.01 (0.95-1.08) | |
| Surgery+adjuvant | 94.0^#^ | 1.05 (1.04-1.06)* | | 94.7^#^ | 1.05 (1.01-1.08)* | | 94.7^#^ | 1.03 (1.02-1.04)* | | 90.7^#^ | 1.08 (1.05-1.11)* | |
| Surgery+adjuvant+  neoadjuvant | 81.4^#^ | 1.05 (1.03-1.07)* | | 84.4^#^ | 1.04 (1.00-1.09) | | 81.4^#^ | 1.03 (1.01-1.04)* | | 76.9^#^ | 1.11 (1.05-1.17)* | |
| Other therapy only^c^ | 38.5^#^ | 0.71 (0.68-0.74)* | | 46.8^#^ | 0.77 (0.68-0.87)* | | 34.7^#^ | 0.65 (0.61-0.68)* | | 44.1^#^ | 0.83 (0.78-0.88)* | |
| **Treatment delay (days)** |  |  | |  |  | |  |  | |  |  | |
| ≤30 | 89.0 | Reference | | 91.0 | Reference | | 89.8 | Reference | | 84.6 | Reference | |
| 31-60 | 90.0 | 1.01 (1.01-1.02)* | | 90.7 | 1.00 (0.99-1.02) | | 91.2^#^ | 1.02 (1.01-1.02)* | | 84.0 | 0.99 (0.98-1.01) | |
| 61-90 | 87.4 | 1.01 (0.99-1.02) | | 85.8 | 0.98 (0.92-1.03) | | 88.4 | 1.01 (0.99-1.03) | | 84.3 | 1.01 (0.97-1.05) | |
| >90 | 80.1^#^ | 1.02 (0.99-1.04) | | 80.9^#^ | 0.99 (0.92-1.07) | | 80.8^#^ | 1.02 (1.00-1.04) | | 77.0^#^ | 1.02 (0.97-1.08) | |

RR: risk ratio; CI: confidence interval; BC: breast cancer. ^#^*p*<0.05 for unadjusted analysis; **p*<0.05 for adjusted analysis

^a^Biomarker expression level was available from 2011.

^b^All adjusted analyses adjusted for year at diagnosis and age at diagnosis. For the adjusted analyses involving cancer stage and histological type, they were mutually adjusted. For the adjusted analyses involving biomarker expression level and any cancer treatment, they were adjusted for cancer stage and histological type. For the adjusted analyses involving treatment type and treatment delay, they were mutually adjusted and adjusted for cancer stage and histological type.

^c^Includes only receiving chemotherapy, radiotherapy, or hormone therapy.

**Supplemental Table 3. Crude risk ratios for the unadjusted association between lifestyle characteristics and five-year overall survival**

| Lifestyle Characteristics | All BC | | Young adult BC | | | Middle-aged BC | | | Elderly BC | | |  |
| --- | --- | --- | --- | --- | --- | --- | --- | --- | --- | --- | --- | --- |
|  | Five-year survival (%) | Crude RR  (95% CI) | | Five-year survival (%) | Crude RR  (95% CI) | | Five-year survival (%) | Crude RR  (95% CI) | | Five-year survival (%) | Crude RR  (95% CI) | |
| **Body mass index (kg/m^2^)** |  |  | |  |  | |  |  | |  |  | |
| Normal (18.5-23.9) | 87.8 | Reference | | 91.0 | Reference | | 89.6 | Reference | | 75.4 | Reference | |
| Underweight (<18.5) | 79.8 | 0.91 (0.89-0.93)* | | 90.9 | 1.00 (0.97-1.03) | | 80.7 | 0.91 (0.88-0.93)* | | 54.8 | 0.73 (0.65-0.82)* | |
| Overweight (24-26.9) | 87.5 | 0.99 (0.99-1.00) | | 90.1 | 0.99 (0.96-1.02) | | 89.6 | 1.00 (0.99-1.01) | | 80.1 | 1.06 (1.03-1.09)* | |
| Slightly obese (27-29.9) | 87.1 | 0.99 (0.98-1.00) | | 87.4 | 0.96 (0.92-1.01) | | 89.2 | 0.99 (0.98-1.01) | | 81.7 | 1.08 (1.04-1.11)* | |
| Moderately obese (30-34.9) | 85.9 | 0.98 (0.96-1.01) | | 94.7 | 1.03 (0.99-1.07) | | 87.7 | 0.98 (0.96-0.99)* | | 79.3 | 1.05 (1.00-1.09) | |
| Severely obese (≥35) | 81.7 | 0.93 (0.90-0.96)* | | 82.1 | 0.90 (0.80-1.02) | | 83.9 | 0.93 (0.90-0.97)* | | 74.9 | 1.00 (0.92-1.09) | |
| **Cigarette smoking** |  |  | |  |  | |  |  | |  |  | |
| Never | 87.1 | Reference | | 90.6 | Reference | | 89.1 | Reference | | 78.0 | Reference | |
| Former | 85.8 | 0.98 (0.94-1.03) | | 89.7 | 0.99 (0.91-1.08) | | 88.3 | 0.99 (0.95-1.04) | | 71.2 | 0.91 (0.78-1.07) | |
| Current | 85.9 | 0.99 (0.97-1.01) | | 90.8 | 1.00 (0.96-1.04) | | 86.5 | 0.97 (0.95-0.99)* | | 68.5 | 0.88 (0.77-1.00) | |
| **Alcohol drinking** |  |  | |  |  | |  |  | |  |  | |
| Never | 87.0 | Reference | | 90.6 | Reference | | 89.0 | Reference | | 77.6 | Reference | |
| Former | 80.8 | 0.93 (0.87-1.00) | | 88.2 | 0.97 (0.82-1.16) | | 80.5 | 0.91 (0.84-0.98)* | | 77.8 | 1.00 (0.82-1.23) | |
| Current (casual) | 89.6 | 1.03 (1.01-1.05)* | | 92.3 | 1.02 (0.98-1.06) | | 89.7 | 1.01 (0.99-1.03) | | 85.8 | 1.11 (1.04-1.18)* | |
| Current (habitual) | 87.5 | 1.01 (0.97-1.04) | | 85.2 | 0.94 (0.84-1.05) | | 88.8 | 1.00 (0.96-1.04) | | 76.7 | 0.99 (0.81-1.20) | |

RR: risk ratio; CI: confidence interval; BC: breast cancer; **p*<0.05

**Supplemental Table 4. Associations between lifestyle characteristics and five-year breast cancer-specific survival of female invasive breast cancer patients**

| Lifestyle Characteristics | All BC | | Young adult BC | | | Middle-aged BC | | | Elderly BC | | |  |
| --- | --- | --- | --- | --- | --- | --- | --- | --- | --- | --- | --- | --- |
|  | Five-year survival (%) | Adjusted RR (95% CI)^a^ | | Five-year survival (%) | Adjusted RR (95% CI)^a^ | | Five-year survival (%) | Adjusted RR (95% CI)^a^ | | Five-year survival (%) | Adjusted RR (95% CI)^a^ | |
| **Body mass index (kg/m^2^)** |  |  | |  |  | |  |  | |  |  | |
| Normal (18.5-23.9) | 90.1 | Reference | | 91.5 | Reference | | 90.9 | Reference | | 84.0 | Reference | |
| Underweight (<18.5) | 83.0^#^ | 0.96 (0.95-0.98)* | | 91.4 | 1.00 (0.97-1.03) | | 82.5^#^ | 0.97 (0.95-0.99)* | | 70.3^#^ | 0.91 (0.85-0.99)* | |
| Overweight (24-26.9) | 90.3 | 1.01 (1.00-1.02) | | 90.3 | 0.99 (0.97-1.02) | | 91.1 | 1.01 (1.00-1.01) | | 88.0^#^ | 1.02 (1.00-1.04) | |
| Slightly obese (27-29.9) | 90.2 | 1.01 (1.00-1.02) | | 87.6 | 0.97 (0.93-1.01) | | 90.8 | 1.01 (1.00-1.02) | | 89.3^#^ | 1.04 (1.01-1.06)* | |
| Moderately obese (30-34.9) | 89.7 | 1.01 (1.00-1.02) | | 94.7 | 1.04 (1.00-1.08) | | 89.6 | 1.00 (0.98-1.01) | | 88.9^#^ | 1.04 (1.01-1.07)* | |
| Severely obese (≥35) | 86.9^#^ | 0.98 (0.96-1.01) | | 83.9 | 0.95 (0.86-1.05) | | 87.9 | 0.97 (0.94-1.00) | | 85.0 | 1.01 (0.95-1.07) | |
| **Cigarette smoking** |  |  | |  |  | |  |  | |  |  | |
| Never | 89.8 | Reference | | 91.0 | Reference | | 90.5 | Reference | | 86.6 | Reference | |
| Former | 87.9 | 1.00 (0.97-1.03) | | 89.7 | 0.99 (0.92-1.07) | | 89.9 | 1.02 (0.98-1.05) | | 78.0 | 0.95 (0.85-1.05) | |
| Current | 88.2 | 0.98 (0.96-0.99)* | | 92.1 | 0.99 (0.95-1.03) | | 88.5 | 0.98 (0.96-1.00) | | 76.9^#^ | 0.92 (0.84-1.01) | |
| **Alcohol drinking** |  |  | |  |  | |  |  | |  |  | |
| Never | 89.7 | Reference | | 91.1 | Reference | | 90.5 | Reference | | 86.3 | Reference | |
| Former | 83.4^#^ | 0.98 (0.94-1.03) | | 88.2 | 1.04 (0.94-1.14) | | 83.9 | 0.98 (0.93-1.03) | | 77.8 | 0.95 (0.81-1.12) | |
| Current (casual) | 91.1 | 1.00 (0.99-1.02) | | 92.9 | 1.02 (0.98-1.06) | | 90.9 | 1.00 (0.98-1.01) | | 90.1 | 1.02 (0.98-1.07) | |
| Current (habitual) | 89.6 | 1.01 (0.98-1.04) | | 85.2 | 0.94 (0.85-1.04) | | 90.9 | 1.01 (0.98-1.04) | | 83.3 | 1.03 (0.89-1.19) | |

RR: risk ratio; CI: confidence interval; BC: breast cancer. ^#^*p*<0.05 for unadjusted analysis; **p*<0.05 for adjusted analysis

^a^For the adjusted analysis involving body mass index, cigarette smoking, and alcohol drinking, they were mutually adjusted and adjusted for year at diagnosis, age at diagnosis, cancer stage, and histological type.

**Supplemental Figure 1. Five-year survival of female invasive breast cancer patients by age at diagnosis**


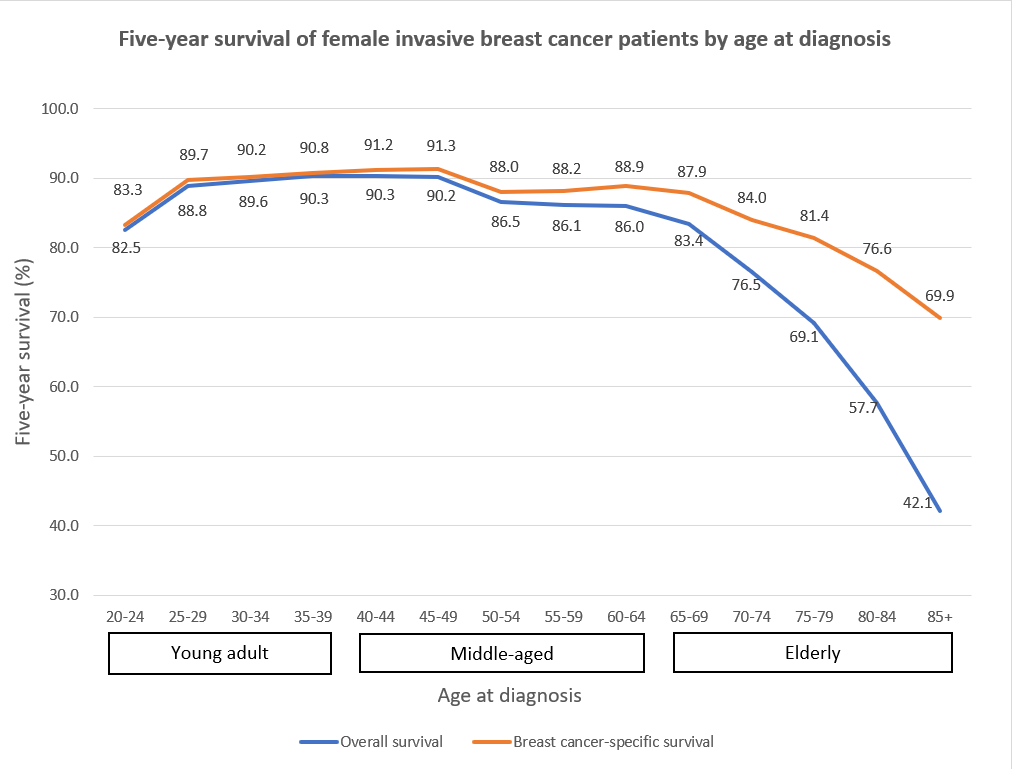

Supplement: Supplementary file 1 — Supplementary material 1 (DOCX 82.8 kb) [file 10549_2024_7280_MOESM1_ESM.docx]
